# Factors associated with comorbidity of diarrhoea and acute respiratory infections among children under five years in Ghana

**DOI:** 10.1371/journal.pone.0271685

**Published:** 2022-07-21

**Authors:** Grace Frempong Afrifa-Anane, Frank Kyei-Arthur, Martin Wiredu Agyekum, Ernest Kwabena Afrifa-Anane

**Affiliations:** 1 Department of Environment and Public Health, University of Environment and Sustainable Development, Somanya, Eastern Region, Ghana; 2 Institute for Educational Research and Innovation Studies, University of Education, Winneba, Ghana; 3 Akrofi-Christaller Institute of Theology, Mission and Culture, Akropong-Akuapem, Ghana; Boston Children’s Hospital, UNITED STATES

## Abstract

**Introduction:**

Globally, childhood mortality is an important public health concern. In Ghana, both diarrhoea and acute respiratory infections (ARIs) are among the top five causes of morbidity and mortality among children under five years old (CU5). Yet, there is a paucity of studies on the comorbidity of diarrhoea and ARIs in CU5 in Ghana.

**Aim:**

This study sought to examine factors associated with comorbidity of diarrhoea and ARIs among CU5 in Ghana.

**Methods:**

The Ghana Demographic and Health Survey (GDHS) 2014 was used for this study. A total of 932 CU5 who had at least one morbidity were included in the study. Binary logistic regression was used to predict the factors associated with comorbidity among CU5.

**Results:**

The prevalence of comorbidity of diarrhoea and ARI among CU5 was 11%. Factors including unimproved source of water, unimproved main floor material, age of child, and initiation of breastmilk were significantly associated with comorbidity of diarrhea and ARI. Improved source of water (AOR = 0.42; 95% CI = 0.22–0.78; p = 0.01) reduces the likelihood of having comorbidity than unimproved source of water. Children aged 36–47 months were less likely (AOR = 0.36; 95% CI = 0.14–0.93; p = 0.04) to have comorbidity than those aged 48–59 months. Also, improved floor materials (AOR = 0.45; 95% CI = 0.22–0.95; p = 0.03) reduces the likelihood of having comorbidity than unimproved floor materials. Children breastfed within the first day of birth were more likely (AOR = 1.66; 95% CI = 1.01–0.2.72; p = 0.04) to have comorbidity than those breastfed immediately after birth.

**Conclusion:**

Policymakers and health practitioners should consider risk factors such as age of child, initiation of breastfeeding, unimproved floor material, and unimproved water supply in the design of interventions to reduce morbidity and mortality associated with comorbidity of diarrhoea and ARI among CU5.

## Introduction

Globally, childhood mortality is an important public health concern and thus, the Sustainable Development Goal (SDG) 3 aims to reduce under-5 mortality rate to as low as 25 per 1000 live births by 2030 [1, 2]. According to the United Nations Inter-agency Group for Child Mortality Estimation (UN IGME) [3], the world has made remarkable progress in reducing under-five mortality rate from 93 per 1,000 live births in 1990 to 38 in 2019. Notwithstanding the progress made worldwide, the burden of under-five mortality rate is still high in sub-Saharan Africa (SSA) with 76 deaths per 1,000 live births in 2019. In Ghana, under-five mortality rate was 46 deaths per 1,000 live births in 2019 [3]. SSA is therefore, far from achieving the SDG target 3.2.1 of 25 per 1000 live birth by 2030.

Evidence from the literature has shown that the major cause of mortality in CU5 years is the comorbidity of diseases [4, 5], that is, the simultaneous occurrence of more than one disease in the same child either at the same time or in some causal sequence. Diarrhoea and ARIs constitute the main burden of morbidity and mortality in CU5 in developing countries [6].

The occurrence of diarrhoea can result in pneumonia, particularly among malnourished children due to weakened immune system. Similarly, malnourished children have a higher susceptibility to developing diarrhoea, thus, creating a vicious cycle [7, 8]. Globally, diarrhoea (the passage of three or more loose or liquid stools per day), is estimated to affect 1.7 billion CU5 annually, with about 525,000 of them dying from the disease [9]. ARI is caused by viruses or bacteria that affect the upper respiratory tract (nose, vocal cords, and ears) and lower respiratory tract (trachea, bronchi, bronchioles, and alveoli), and it accounted for 15% of deaths in CU5 in 2017. The highest proportion of ARI-associated deaths occurred in sub-Saharan Africa and Asia [10].

Studies in Asia and some sub-Saharan African countries have indicated that several socio-economic, demographic, and environmental factors including, educational level of mothers, mother’s age, household wealth, and unimproved sanitation facilities are associated with comorbidity of diarrhoea and ARI [5, 11–13]. For example, a study carried out by Mutala and Mulatya [5] in Kenya reported that the risk of comorbidity of diarrhoea and ARI among CU5 was lower among older caregivers, older children and high wealth quintile households.

In Ghana, both diarrhoea and ARIs are among the top five causes of morbidity and mortality among CU5 [14]. The high rate of diarrhoea and ARI morbidities in CU5 have been linked to the slow pace in the decline in under-five mortality from 111 per 1,000 live births in 1988 to 56 per 1000 live births in 2017 [4]. However, there is a paucity of studies on the comorbidity of diarrhoea and ARI in CU5 in Ghana [8, 15]. Previous studies in Ghana have examined diarrhoea and ARIs independently without focusing on their simultaneous occurrence [4, 16, 17]. Some of these studies were also carried out in selected districts and health facilities in Ghana, hence were not representative of children under five [18]. Meanwhile, few studies such as that of Fenn et al. [8] in Northern Ghana found that the comorbidity of diarrhoea and pneumonia is more prevalent than other comorbidity conditions such as diarrhoea and measles. Amugsi et al.’s [15] study in Ghana also adds that children older than 5 months, and those from poor households were more likely to experience diarrhoea and ARI.

This calls for an in-depth analysis of the factors associated with the comorbidity of diarrhoea and ARI to gain insight into this health problem to develop strategies to combat it. Therefore, this study sought to examine the prevalence and risk factors associated with comorbidity of diarrhoea and ARI in CU5 in Ghana. Findings from this study will provide a holistic understanding of the co-occurrence of the two conditions and, thus, help policymakers and health practitioners to develop integrated public health interventions to help reduce morbidity and mortality in CU5.

## Materials and methods

### Study setting

Ghana is located in the Western part of Africa with an estimated 30.9 million population. It is bounded by Burkina Faso to the North, the Gulf of Guinea to the South, Togo to the East, and Cote d’Ivoire to the West. Ghana has 16 administrative regions. A little over half of the households (53.4%) in Ghana have a place for washing hands, while about two-fifth of households (39.5%) have water, soap and a cleaning agent other than soap at a location for washing hands [19]. As of 2021, most households (92%) in Ghana had access to an improved source of drinking water. However, about 18% of households had no access to toilet facilities, with most household members defecating in the open field, gutter or bush [20].

In addition, studies have shown that diarrhoea and ARI are more prevalent during the rainy seasons [18, 21]. A plausible explanation is that rain contaminates water bodies with human excreta that are indiscriminately disposed of during the rainy season, which increases the risk of waterborne diseases, including diarrhoea. Also, during the rainy season, most people spend much time indoors, so when one is infected with ARI, it could easily be transmitted to others, including CU5 [21].

There are also disparities in the proximity and availability of primary healthcare facilities to residents. Generally, more than half of residents (52%) live within a kilometre to reach a primary healthcare facility (private and public hospitals, clinics/health centres, and community-based health planning and services, etc.). However, about 9 out of 10 residents in urban areas live within a kilometre to reach a primary healthcare facility compared to about two-fifths of residents in rural areas (35%) [22].

### Data collection

This study used secondary data from the 2014 Ghana Demographic Health Survey (GDHS). The GDHS is a nationally representative sample survey that is conducted every five years by Ghana Statistical Service. The 2014 GDHS is the sixth round in the series. A two-stage sample design was employed during the data collection. This involved selection of clusters consisting of the enumeration areas (EAs) in both rural and urban areas for the first stage and the second stage involved systematic sampling of households from the selected EAs for the survey. Listing was done in all selected households across the country and eligible women (15–49 years) and men (15–54 years) were interviewed. In addition, mothers who gave birth five years preceding the survey responded to the questions relating to their CU5. We limited our analysis to CU5 who experienced either diarrhoea or ARI. A sample size of 932 CU5 was used in this study.

### Measurement

#### Dependent variable

The dependent variable for the study was comorbidity. This was computed by combining two variables, namely: Diarrhoea and ARI. First, ARI was computed from two questions. During the survey, mothers with CU5 were asked if “(name) had an illness with a cough at any time in the last 2 weeks preceding the survey” and “when (name) had an illness with a cough did he or she breathe faster than usual with short, rapid breaths or have difficulty breathing in the last 2 weeks preceding the survey”. Mothers who indicated that their child(ren) had a cough in the last 2 weeks preceding the survey responded to the second question. Hence, CU5 who had a cough within the last 2 weeks preceding the survey and breathed faster than usual with short, rapid breaths or had difficulty in breathing were classified as having ARI, and otherwise as not having ARI. In addition, diarrhoea prevalence was estimated from children born five years preceding the survey. During the survey, mothers were asked if their child(ren) had diarrhoea in the last 2 weeks preceding the survey. Children under five years who had any episode of diarrhoea were classified as “Yes”, otherwise they were classified as “No”.

A child was classified as having comorbidy if the child had both ARI and diarrhoea. Children who had either ARI or diarrhoea were classified as having no comorbidity. All CU5 who had no experience of diarrhoea or ARI were excluded from the analysis.

#### Independent variables

The independent variables consisted of maternal characteristics, child-related factors, and household characteristics. These variables were selected based on factors identified in the literature as predictors of ARI and diarrhoea [1, 4, 5, 11–13]. The maternal characteristics included the age of mother (15–19 years, 20–24 years, 25–29 years, 30–34 years, 35–39 years, 40–44 years, and 45–49 years), place of residence of mother (urban, and rural), educational level of mother (no education, primary, secondary, and higher education), marital status of mother (never in a union, currently married, and formerly married) and children ever born (1, 2–4, and 5 and above).

Child-related factors included sex of child (male and female), age of child (0–5 months, 6–11 months, 12–23 months, 24–35 months, 36–47 months, and 48–59 months), health insurance (Yes (those with valid insurance) and No (those without insuance insuance and those with NHIS insurance card that had expired), initiation of breastfeeding (immediately after birth, within the first day of birth, and after the first day of birth), postnatal (Yes (mothers who went to the health facility for postnatal service after delivery) and No (mothers who did not visit the health facility for postnatal service after delivery), stunting (Yes (children whose z-score fell below -2 standard deviations from the mean) and No (children whose z-score did not fall below -2 standard deviations from the mean), wasting (Yes (children whose z-score fell below -2 standard deviations from the mean) and No (children whose z-score did not fall below -2 standard deviations from the mean), child sleeping in a mosquito net (some children, all children, and no child), and birth order which was measured as a continuous variable.

Household characteristics included sources of drinking water, sanitation, floor materials, wealth index and number of children in households. Drinking water was categorised as improved (boreholes, piped water or tube wells, protected dug wells, protected springs, tanker-truck, rain water, and packaged water) and unimproved (unprotected springs, unprotected dug wells, and surface water collected directly from a river, dam, lake, pond, stream, canal and irrigation channels). Similarly, sanitation was grouped as improved (flush/pour flush, toilet, pit latrine with slab, ventilated improved latrine, and composting toilet) and unimproved (pit latrines without a slab or platform, hanging latrines or bucket latrines). In addition, floor material was classified as improved (made up of cement, ceramic tiles, vinyl asphalt strips, parquet, polished wood,) and unimproved (earth, sand, dung, wood planks, palm, bamboo were considered unimproved). Wealth index was categorised as poorest, poorer, middle, richer, and richest. Lastly, the number of children in households was classified as 1, 2, 3, and 4 and above.

#### Statistical analysis

The data were analysed with Stata version 15. The data were weighted and primary sampling unit and sample strata for sampling errors were used to create the svy command to account for clusters or stratification to make it representative and to provide a better statistical estimate. The charactistics of respondents were described using descriptive statistics. At the bivariate level of analysis, Pearson chi-square test was used to determine the association between comorbidity and each of the independent variables. For the multivariate analysis, binary logistic regression model was used to examine the relationship between comorbidity and the independent variables. Two models (Model I and II) were built. Model I examined the relationship between comorbidity and each individual independent variable separately, while Model II examined the relationship between comorbidity and all the independent variables together. In Model II, the Enter method was used where all the independent variables were entered into the Model at a single step. A p-value < 0.05 was considered to be significant.

## Results

### Comorbidity of children under age 5

**Fig 1:**
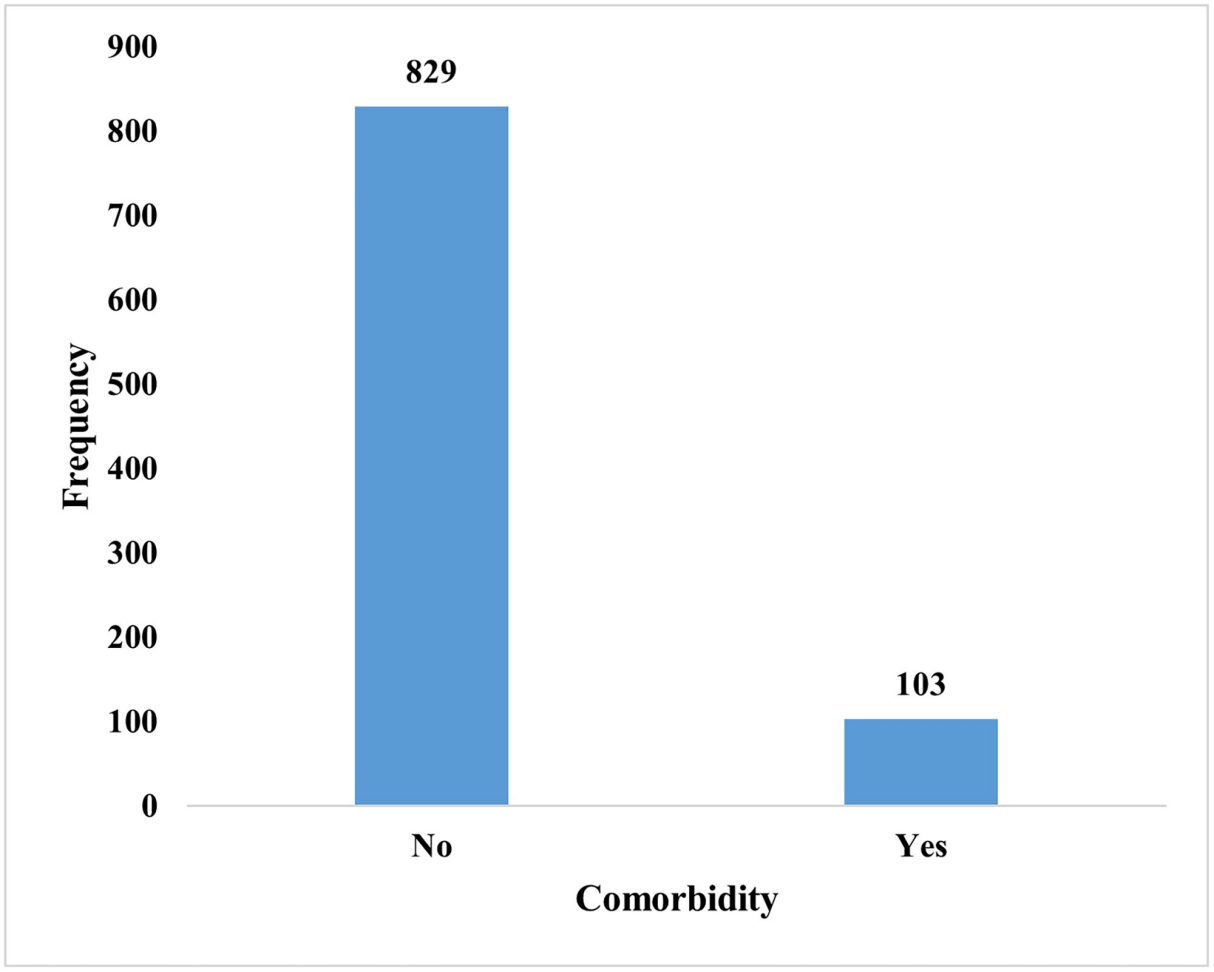
Comorbidity of diarrhoea and ARIs among CU5.

The result in Fig 1 indicates that for every ten children under the age of five years with either diarrhoea or ARI, one had (11%) a comorbid condition.

### Maternal characteristics

The age structure of the mothers to the children under age five with either diarrhoea or ARI shows a bell shape. The highest proportion (25%) of the mothers were aged 25–29 years, this was followed by those aged 30–34 years (24%) and a few (2.9%) were aged 40–49 (Table 1). More than two-thirds (61.6%) of the mothers resided in rural areas. In terms of wealth status, slightly less than one-third (32.3%) was in the poorest wealth quintile, 23.5% was in the poorer wealth quintile and 22.0% was in the middle wealth quintile. The higher proportion of the mothers had some form of formal education, less than 5% had attained a higher level of education and two-fifths (40%) had secondary level of education. Regarding marital status, eight out of ten of the mothers were currently married and the highest proportion (19.3%) of them had attained a parity of 5–6 children.

**Table 1:** Description of maternal characteristics.

Maternal characteristics		Comorbidity		Chi-square
		No	Yes	
	Frequency (Percentage)	Frequency (Percentage)	Frequency (Percentage)	
**Age of mother**				2.16
15–19	50 (5.36)	44 (88.00)	6 (12.00)	
20–24	168 (18.03)	153 (91.07)	15 (8.93)	
25–29	233 (25.00)	203 (87.12)	30 (12.88)	
30–34	224 (24.03)	201 (89.73)	23 (10.27)	
35–39	154 (16.52)	138 (89.61)	16 (10.39)	
40–44	76 (8.15)	66 (86.84)	10 (13.16)	
45–49	27 (2.90)	24 (88.89)	3 (11.11)	
**Place of residence**				0.59
Urban	358 (38.41)	322 (89.94)	36 (10.06)	
Rural	574 (61.59)	507 (88.33)	67 (11.67)	
**Wealth index**				1.95
Poorest	301 (32.30)	268 (89.04)	33 (10.96)	
Poorer	219 (23.50)	193 (88.13)	26 (11.87)	
Middle	205 (22.00)	179 (87.32)	26 (12.68)	
Richer	122 (13.09)	112 (91.80)	10 (8.20)	
Richest	85 (9.12)	77 (90.59)	8 (9.41)	
**Educational level**				1.50
No education	336 (36.05)	297 (88.39)	39 (11.61)	
Primary	197 (21.14)	177 (89.85)	20 (10.15)	
Secondary	375 (40.24)	332 (88.53)	43 (11.47)	
Higher	24 (2.58)	23 (95.83)	1 (4.17)	
**Marital status**				0.65
Never in union	79 (8.48)	72 (91.14)	7 (8.86)	
Currently married	792 (84.98)	704 (88.89)	88 (11.11)	
Formerly married	61 (6.55)	53 (86.89)	8 (13.11)	
**Children ever born**				0.09
1	173 (18.56)	153 (88.44)	20 (11.56)	
2–4	483 (51.82)	431 (89.23)	52 (10.77)	
5 and above	276 (29.62)	245 (88.77)	31 (11.23)	
**Total**	**932 (100.00)**			

* = p < .1,

** = p < .05,

*** = p < .01

Source: GDHS, 2014.

### Child characteristics

The data indicates that 54.7% of the children were males (Table 2). The highest proportion (27.6%) of them were aged 12 to 23 months and the least proportion (6.33%) was those aged 0 to 5 months. The prevalence of comorbidity was highest among children aged 48–59 months but lowest among those aged 36 to 47 months (p = 0.02). One-third (29.6%) of the children had no health insurance. Though all the children were breastfed, there are differences in the time of initiation: 48.5% were introduced to breastfeeding right after birth, 40.7% later within the first day of birth, and 10.8% were initiated after the first day of birth. After birth, one-fifth (22.1%) of the children did not visit the health facility for child health care services. Regarding their health status, more than half (58.6) of the children had no stunting and 67.6% had no wasting. Finally, 47.5% of all the children slept in a mosquito net and the mean birth order was 3.41.

**Table 2:** Description of child-related characteristics.

Child-related characteristics		Comorbidity		Chi-square
		No	Yes	
	Frequency (Percentage)	Frequency (Percentage)	Frequency (Percentage)	
**Sex of child**				0.31
Male	510 (54.72)	451 (88.43)	59 (11.57)	
Female	422 (45.28)	378 (89.57)	44 (10.43)	
**Age of child (in months)**				13.74 ***
0–5	59 (6.33)	51 (86.44)	8 (13.56)	
6–11	127 (13.63)	110 (86.61)	17 (13.39)	
12–23	257 (27.57)	220 (85.60)	37 (14.40)	
24–35	245 (26.29)	229 (93.47)	16 (6.53)	
36–47	138 (14.81)	129 (93.48)	9 (6.52)	
48–59	106 (11.37)	90 (84.91)	16 (15.09)	
**Health insurance**				1.06
No	276 (29.61)	241 (87.32)	35 (12.68)	
Yes	656 (70.39)	588 (89.63)	68 (10.37)	
**Initiation of breastfeeding**				4.53
Immediately after birth	452 (48.50)	412 (91.15)	40 (8.85)	
Within the first day of birth	379 (40.67)	328 (86.54)	51 (13.46)	
After the first day of birth	101 (10.84)	89 (88.12)	12 (11.88)	
**Postnatal**				0.01
No	206 (22.10)	183 (88.83)	23 (11.17)	
Yes	726 (77.90)	646 (88.98)	80 (11.02)	
**Stunting**				2.64
No	546 (58.58)	478 (87.55)	68 (12.45)	
Yes	386 (41.42)	351 (90.93)	35 (9.07)	
**Wasting**				0.02
No	630 (67.60)	561 (89.05)	69 (10.95)	
Yes	302 (32.40)	268 (88.74)	34 (11.26)	
**CU5 who slept in mosquito net**				4.06
Some children	61 (6.59)	57 (93.44)	4 (6.56)	
All children	440 (47.52)	383 (87.05)	57 (12.95)	
No child	425 (45.90)	385 (90.59)	40 (9.41)	
**Birth order**	Minimum, Maximum (mean, SD)	Spearman	Probability	
	1, 13 (3.41, 2.18)	0.03	0.39	
**Total**	**932 (100.00)**			

SD = Standard deviation;

* = p < .1,

** = p < .05,

*** = p < .01

Source: GDHS, 2014.

### Household characteristics

Nearly, one-third (28.5%) of the households did not have improved water as a source of drinking water, and two-fifths (42.7%) had unimproved sanitation (Table 3). Regarding the type of floor material in households, about nine out of ten households had unimproved floor material. However, the prevalence of comorbidity was higher among households with improved floor materials (17.5%) than those with unimproved materials (p = 0.03). Lastly, almost all households had at least one child in the household.

**Table 3:** Description of household characteristics.

Household characteristics		Comorbidity		Chi-square
		No	Yes	
	Frequency (Percentage)	Frequency (Percentage)	Frequency (Percentage)	
**Source of drinking water**				1.13
Improved water	666 (71.46)	597 (89.64)	69 (10.36)	
Unimproved water	266 (28.54)	232 (87.22)	34 (12.78)	
**Sanitation**				0.05
Improved sanitation	534 (57.30)	476 (89.14)	58 (10.86)	
Unimproved sanitation	398 (42.70)	353 (88.69)	45 (11.31)	
**Floor materials**				4.62 **
Improved materials	97 (10.41)	80 (82.47)	17 (17.53)	
Unimproved materials	835 (89.59)	749 (89.70)	86 (10.30)	
**Number of children in household**				0.96
1	405 (43.45)	363 (89.63)	42 (10.37)	
2	378 (40.56)	332 (87.83)	46 (12.17)	
3	113 (12.12)	101 (89.38)	12 (10.62)	
4 and above	36 (3.86)	33 (91.67)	3 (8.33)	
**Total**	**932 (100.00)**			

* = p < .1,

** = p < .05,

*** = p < .01

Source: GDHS, 2014.

### Logistic regression analyses

We reported the unadjusted (Model I) and adjusted (Model II) odds ratios in Table 4. In Model I, age of child was the only variable which was significantly associated with comorbidity. Age of child remained significant after controlling for other variables (Model II). Children aged between 36–47 months were less likely (AOR = 0.36; 95% CI = 0.14–0.93; p = 0.04) to have comorbidity than those aged 48–59 months.

**Table 4:** Binary logistics regression showing the factors associated with comorbidity.

Variables	Model I	Model II
	COR (95% CI)	AOR (95% CI)
**Age of mother**		
15–19 (RC)		
20–24	0.83 (0.21–3.23)	0.94 (0.20–4.44)
25–29	1.51 (0.44–5.15)	2.04 (0.41–10.04)
30–34	1.16 (0.32–4.12)	1.79 (0.34–9.52)
35–39	0.84 (0.26–3.12)	0.92 (0.17–5.07)
40–44	1.27 (0.34–4.76)	1.31 (0.21–8.03)
45–49	1.26 (0.23–6.74)	1.20 (0.11–12.61)
**Place of residence**		
Urban (RC)		
Rural	1.17 (0.66–2.09)	0.92 (0.46–1.83)
**Wealth quintile**		
Poor (RC)		
Poorer	1.53 (0.60–3.87)	2.03 (0.71–5.80)
Middle	1.15 (0.58–2.28)	1.93 (0.79–4.72)
Richer	0.78 (0.33–1.83)	1.08 (0.34–3.40)
Richest	0.87 (0.29–2.61)	0.91 (0.20–4.17)
**Educational level**		
No education (RC)		
Primary	0.59 (0.27–1.30)	0.46 (0.20–1.06)
Secondary	0.77 (0.40–1.47)	0.70 (0.32–1.53)
Higher	0.25 (0.33–1.92)	0.21 (0.02–2.01)
**Marital status**		
Never in union (RC)		
Currently union/living with a man	1.02 (0.39–2.64)	0.93 (0.30–2.85)
Formerly union/living with a man	1.31 (0.35–5.89)	1.32 (0.31–5.63)
**Source of water**		
Unimproved water (RC)		
Improved water	0.56* (0.31–1.01)	0.42*** (0.22–0.78)
**Sanitation**		
Improved sanitation (RC)		
Unimproved sanitation	1.32 (0.76–2.30)	0.98 (0.57–1.70)
**Main floor materials**		
Unimproved (RC)		
Improved	0.50* (0.24–1.03)	0.45** (0.22–0.95)
**Sex of infant**		
Male (RC)		
Female	0.79 (0.48–1.30)	0.78 (0.48–1.25)
**Age of child**		
0–5 months	1.12 (0.42–3.62)	1.37 (0.48–3.96)
6–11 months	1.11 (0.41–3.04)	0.99 (0.37–2.66)
12–23 months	0.86 (0.41–1.78)	0.94 (0.43–2.02)
24–35 months	0.43 (0.17–1.09)	0.45 (0.18–1.14)
36–47 months	0.36** (0.14–0.93)	0.36** (0.14–0.93)
48–59 months (RC)		
**Initiation of breastfeeding**		
Immediately after birth (RC)		
Within first day	1.66** (1.01–2.72)	1.66** (1.01–2.72)
After first day	1.19 (0.46–3.07)	1.19 (0.46–3.07)
**Children ever born**		
1 (RC)		
2–4	0.82 (0.39–1.72)	0.36 (0.10–1.22)
5 and above	0.75 (0.35–1.60)	0.18 (0.02–1.25)
**Children in the household**		
1 (RC)		
2	1.08 (0.64–1.82)	1.63 (0.86–3.06)
3	0.74 (0.33–1.69)	0.84 (0.33–2.19)
4 and above	0.65 (0.18–2.41)	0.87 (0.20–3.79)
**Stunting**		
No (RC)		
Yes	0.77 (0.43–1.35)	0.60 (0.32–1.13)
**Wasting**		
No (RC)		
Yes	0.91 (0.53–1.57)	1.28 (0.66–2.47)
**Birth order**	1.02 (0.89–1.16)	1.26 (0.92–1.73)
**Health insurance**		
No (RC)		
Yes	0.86 (0.54–1.38)	0.83 (0.49–1.41)
**Sleep in mosquito net**		
Some children (RC)		
All children	1.50 (0.43–5.52)	1.54 (0.36–6.58)
No net in household	1.51 (0.43–5.38)	1.52 (0.38–6.10)
**Postnatal**		
No (RC)		
Yes	0.88 (0.50–1.53)	1.03 (0.54–1.97)
_cons		0.35 (0.05–2.74)

RC = Reference category; COR = Crude odds ratio; AOR = Adjusted odds ratio; CI = Confidence interval;

* = p < .1,

** = p < .05,

*** = p < .01

Source: GDHS, 2014.

In addition, source of water, main floor materials, and initiation of breastfeeding were significantly associated with comorbidity in Model II. Children from households with improved sources of water were less likely (AOR = 0.42; 95% CI = 0.22–0.78; p = 0.01) to have comorbidity than those with unimproved sources of water.

With regards to main floor materials, children from households with improved floor materials were less likely (AOR = 0.45; 95% CI = 0.22–0.95; p = 0.03) to have comorbidity than those with unimproved floor materials. Also, children who were breastfed within the first day of birth were more likely (AOR = 1.66; 95% CI = 1.01–0.2.72; p = 0.04) to develop comorbidity condition compared to infants who were breastfed immediately after birth.

## Discussion

This study examined the prevalence and risk factors associated with comorbidity of diarrhoea and ARI in CU5 in Ghana. This study is relevant as it highlights the factors causing morbidity among children in Ghana. Previous studies in Ghana have not examined the factors contributing to comorbidity of diarrhoea and ARI among CU5 [5, 8]. Our finding therefore reveals factors that contribute to comorbidity among CU5 in Ghana.

Our results show that the prevalence of comorbidity of diarrhoea and ARI in CU5 in Ghana was 11%. This finding is higher than the prevalence found in other sub-Saharan African countries [5, 11]. For instance, Mulatya and Mutuku’s [5] study in Kenya found that the prevalence of comorbidity of diarrhoea and ARI in CU5 was 2.2% (95% CI = 2.0–2.5). Similarly, Adedokun’s [11] study in Nigeria reported that 9% of CU5 experienced comorbidity. The variation in the prevalence of comorbidity of diarrhoea and ARI in this study and previous ones could be attributed to differences in geographical context and risk factors of the study areas such as breastfeeding practices, personal hygiene, sanitation and access to clean drinking water. This high prevalence of 11% of children with comorbidity has implications for Ghana in relation to attaining the Sustainable Development Goal 3 (target 3.2) of ending preventable deaths of newborns and children under 5 years of age. It may partly explain why Ghana is making little progress towards the reduction of under-five mortality [19]. Health policy makers and practitioners need to implement health strategies which focus on reducing comorbidity of diarrhoea and ARIs to attain a decline in under-five mortality.

Our finding also indicates a higher likelihood for children from households with unimproved sources of water to have comorbid conditions compared to those whose households accessed water from improved sources. This finding is similar to a study carried out by Adedokun [11] in Nigeria which reported that children of women who obtained water from improved sources were less likely to experience morbidity compared to children whose mothers obtained water from unimproved sources. Adedokun [11] explained that accessing water from unimproved sources increases the risk of contamination and thus, exposes children who consume such water to water-borne diseases. In Ghana, it was reported that only 44.3% of Ghanaians had access to improved drinking water in 2017 [23]. Though this is high, it is still far below achieving the Global target 6.1 of the SDG 6 [23]. To help reduce comorbidity of diarrhoea and ARI in Ghana, health policy makers should design appropriate startegies to improve source of water or water supply at the household level.

With regards to main floor materials, children from households with improved floor materials were less likely to have comorbid condition, compared to those with unimproved floor materials. This finding corroborates with a study carried out by Woldemicael [24] in Eritrea which found a significant association between unimproved floor materials and childhood illness. Floors made of unimproved materials can serve as breeding grounds for disease-causing agents [25]. However, a study by Duah et al. [1], showed no significant association between the main floor material and comordity among CU5 in Ghana. Though diarrhoea is a common variable in this study and that of Duah et al. [1], the difference in the findings may be attributed to the latter study’s use of anaemia which is more related to nutritional defficiency compared to the use of ARI which is a respiratory disease. In spite of this, our finding of an association with the type of floor material and comorbid condition plausibly suggests the vulnerability of CU5 to disease-causing organisms which may be found on surfaces of unimproved floor materials they have regular contact with.

Regarding the age of a child, children aged 36–47 months were less likely to contract comorbidity of diarrhoea and ARI compared to those aged 48–59 months. This implies that the burden of comorbidity of diarrhoea and ARI is higher in older age groups. There may be differences in age-cohort risk exposures as care for older children may be minimal compared to younger children.

The findings further showed that children who were breastfed later within the first day of birth were more likely to develop comorbidity condition compared to children breastfed immediately after birth. The World Health Organisation recommends early initiation of breastfeeding (i.e. breastfeeding within one hour of birth) since it protects infants against diseases (such as diarrhoea and pneumonia) and reduces neonatal mortality and morbidity [26–29]. The protection early initiation of breastfeeding provides against infant illnesses and neonatal morbidity could explain why children who were breastfed immediately after birth had a lower risk of contracting comorbidity of diarrhoea and ARI than children breastfed later within the first day of birth.

### Limitation

The study had some limitations. First, the incidence of diarrhoea and ARI were self-reported and it could have been misreported. Second, the study was cross-sectional and we, therefore, cannot assume causality between the variables of interest. Third, the 2014 GDHS data used for this study is about 8 years old although it is the current data available. Fourth, this study focused on CU5 who experienced either diarrhoea, ARI or both. Therefore, the results of the study cannot be generalised to all CU5. Lastly, some of the variables were recall and thus, there may be lapses in the response from mothers. Despite these limitations, this study provides policymakers and health practitioners with information on the risk factors associated with comorbidity among CU5 in Ghana.

## Conclusion

This study indicates that CU5 in Ghana are exposed to the risk of comorbidity, with maternal characteristics (early initiation of breastfeeding), child characteristics (age of child) and household characteristics (source of drinking water and the main floor material) playing a significant role. Policymakers and health practitioners should design appropriate interventions to help reduce comorbidity of diarrhoea and ARI among CU5 in Ghana. Risk factors such as age of child, initiation of breastfeeding, unimproved floor material, and unimproved water supply should be considered when designing the interventions.

## Supporting information

S1 Appendix Comorbidity of diarrhoea and ARI. (XLSX)
